# Hyper-Zagreb index in fuzzy environment and its application

**DOI:** 10.1016/j.heliyon.2024.e36110

**Published:** 2024-08-15

**Authors:** Sk Rabiul Islam, Bandar Bin Mohsin, Madhumangal Pal

**Affiliations:** a Department of Mathematics, Brainware University, 398, Ramkrishnapur Road, Jagadighata Market, Barasat, Kolkata-700125, India; b Department of Applied Mathematics, Vidyasagar University, Midnapore 721102, India; c Department of Mathematics, College of Science, King Saud University, P.O. Box 2455, Riyadh 11451, Saudi Arabia; d Department of Mathematics and Innovation, Saveetha School of Engineering, Chennai 602105, Tamilnadu, India

**Keywords:** 05C09, 05C72, Fuzzy graph, Topological indices, First Zagreb index, Second Zagreb index, Hyper-Zagreb index

## Abstract

The Zagreb indices (ZIs) are important graph invariants that are used extensively in many different fields in mathematics and chemistry, such as network theory, spectral graph theory, fuzzy graph theory (FGT) and molecular chemistry, etc. The hyper-ZI is introduced especially for fuzzy graphs (FGs) in this study. The study computes this index's bounds for a variety of FG types, including paths, cycles, stars, complete FGs and partial fuzzy subgraphs. It is shown that isomorphic FGs produce the same values for this index. Moreover, interesting connections are established between the hyper-ZI and the second ZI for FGs. Moreover, bounds on this index are found for the following operations: direct product, Cartesian product, composition, join, union, strong product and semi-strong product of two FGs. In the end, the effectiveness of this index is compared with three other topological indices: hyper-ZI for crisp graphs, first ZI for FGs and F-index for FGs, in an analysis of the crime “Murder” in India. While the hyper-ZI for FGs, first ZI for FGs and F-index for FGs yield similar outcomes, the hyper-ZI for FGs demonstrates superior realism in detecting crimes in India compared to its crisp graph counterpart.

## 1 Introduction

### 1.1 Research background

Fuzzy graph theory marks a notable departure from traditional graph theory by incorporating the notion of fuzziness into graph structures, thus allowing for the representation of imprecise and uncertain information. The seminal work of Zadeh [60] (1965) established the groundwork for fuzzy sets, which introduced the revolutionary concept of degrees of membership. This innovation enabled the modeling of uncertainty and vagueness within mathematical frameworks. Lee et al. [27] present a comprehensive comparison of different types of fuzzy sets, specifically interval-valued fuzzy sets, intuitionistic fuzzy sets, bipolar fuzzy sets, etc. Building upon this foundation, Rosenfeld [48] (1975) further extended the application of fuzzy sets to graph theory, thereby introducing fuzzy graphs (FGs) as a generalization of classical graphs. FG theory has emerged as a powerful tool for modeling imprecise and uncertain information in various real-world applications. Many applications of FGs are described in [29], [40], [50].

Ananthanarayanan and Lavanya [3] introduced the concept of FG coloring, demonstrating how *α*-cuts can be used for this purpose. Rosyida et al. [49] proposed an innovative approach to evaluate the fuzzy chromatic number of a FG, contributing to the understanding of graph coloring in uncertain environments. Bhutani and Rosenfeld conducted a series of studies, including investigations into strong arcs [4], fuzzy end nodes [5], M-strong FGs [6] and geodesies [7]. These works expanded the understanding of connectivity and paths within FGs. Sunitha and Vijayakumar investigated the complement of FGs [57]. Their contributions enriched the field's understanding of graph properties and structures. Tajdin et al. [58] addressed the computation of fuzzy shortest paths using *α*-cuts, demonstrating practical applications of FGs in network analysis and optimization. Nagoorgani et al. [36] introduced the idea of fuzzy effective distance k-dominating sets, laying the foundation for understanding dominance in FGs. Sahoo and Pal [54] explored intuitionistic competition FGs, adding a competitive aspect to the fuzzy framework. They further extended their study to intuitionistic tolerance FGs [55]. Samanta and Pal [56] introduced k-competition FGs, respectively, providing a comprehensive exploration of the structural aspects of these FG variations. Akram's seminal work [1] studied the concept of bipolar FGs, where each edge and vertex are associated with both positive and negative membership degrees. Rashmanlou et al. [46] investigated the product of bipolar FGs and their degrees, contributing to a deeper understanding of graph operations within the bipolar fuzzy context. Interval-valued FGs were investigated by Akram and Dudek [2] and further extended to interval-valued threshold FGs [41], interval-valued phi-tolerance competition FGs [42] and interval-valued planar FGs [43], shedding light on the implications of interval-valued fuzziness. Mondal et al. [33] explored the utilization of the isometric and antipodal concepts within *m*-polar FGs. Their study delves into the application of these concepts in solving a road network problem, showcasing the effectiveness of this approach. Furthermore, the authors investigated the idea of a generalized *m*-polar planar FG and its practical application [34], as well as the utilization of Interval-valued intuitionistic soft FGs in real-world scenarios [35]. Many works have been done on FGs and related graphs such as covering and pair domination in intuitionistic fuzzy graphs [53], isomorphism of m-polar fuzzy graphs [12], cubic graph [47], completeness and regularity of FS [52], edge coloring of FGs [30], product bipolar FGs [13], etc.

In 1947, Harold Wiener [59] first introduced the Wiener index and it is used to determine the boiling point of paraffin. In 1971, Hosoya [17] introduced the Hosoya index. Followed by those indices Gutman et al. studied some another TIs: ZIs [14], Szeged index [15], Gutman index [16], F-index [11], etc. Randic introduced the Randic index (connectivity index) [44] and hyper-Wiener index [45]. One can read other TIs: PI index [26], AM-GM index [51], etc. Some degree-based [31] and neighborhood degree-based [32] topological index is studied by Mondal et al. One can also study other topological index in [24] like: Harary index, General Zagreb index, Zagreb coindices, multiplicative version of Zagreb indices, general Randic index, Randic co-index, ABC index, Harmonic index, Geometric-arithmetic index, sum connectivity index, augmented Zagreb index, etc.

### 1.2 Motivation

Many results and applications are available for TIs in crisp graphs. Some circumstances cannot be handled using crisp graphs in many real-life problems. In such cases, to handle the problem, those TIs are needed to introduce in FGs. In 2020, Binu, Mathew and Mordeson [9] first defined a topological index “Wiener index” in a FGs. Connectivity index (CI) and average CI for a FG are also proposed by Binu, Mathew and Mordeson [8]. Also, Islam and Pal have been introduced and studied some TIs for FG: First Zagreb index [18], Hyper-Wiener index [19], F-index [20], Hyper-connectivity index [21], Second Zagreb index [22], Multiplicative first ZI [23], etc. Kalathian et al. [25] discussed so many TIs for FGs also. Fang et al. [10] studied connectivity and Wiener index for fuzzy incidence graph. Mufti et al. studied first and second fuzzy ZI [37]. Liaqat et al. [28] Naeem et al. [38] studied connectivity index for intuitionistic FG. Cyclic connectivity index for fuzzy incidence graph [39] is studied by Nazeer et al. Motivated by these articles, in this paper, the hyper-ZI is introduced and studied for FGs.

### 1.3 Novelty of the article

Topological indices (TIs) play a critical role in chemical graph theory, spectral graph theory, network theory, molecular chemistry, FG theory and various other domains. Among these indices, ZIs are particularly noteworthy as degree-based TIs, initially introduced by Gutman and Trinajstic in 1972. ZIs are instrumental in calculating the *π*-electron energy of conjugate systems, rendering them invaluable in network theory, spectral graph theory, molecular chemistry and numerous fields within chemistry and mathematics.

(i) In this study, the hyper-ZI is defined and examined for FGs.

(ii) The article presents the calculated bounds of this index for several types of FGs, including paths, cycles, stars, complete FGs and partial fuzzy subgraphs.

(iii) Notably, it is demonstrated that for isomorphic FGs, the value of this index remains constant.

(iv) Moreover, an intriguing relationship between the second ZI and the hyper-ZI for FGs is elucidated.

(v) The study further establishes bounds for this index in operations involving Cartesian product, composition, join, union, direct product, strong product and semi-strong product of two FGs.

(vi) Additionally, the article concludes with an analysis of the crime “Murder” in India using this index, providing insights into its application in real-world scenarios.

### 1.4 Framework of the article

The article's structure: Section 2 provides some basic definitions. In section 3, hyper-ZI has been defined and provided some bounds for several FGs: path, cycle, star, partial fuzzy subgraph, complete FG, isomorphic FGs, etc. Also, some exciting relations have been established between the second-ZI and hyper-ZI for a FG. Bounds of this index for the Cartesian product, composition, join, union, direct product, strong product and semi-strong product of two FGs have been established in section 4. In section 5, the crime “Murder” in states of India has been analyzed by this index.

## 2 Preliminaries

In 1975, Rosenfeld introduced the concept of FG. The FG is defined as:

Definition 2.1 [23], [40] A FG is a pair, G=(V,E), where *V* is called vertex set of the FG with vertex membership function ξ:V→[0,1] and *E* is called edge set with edge membership function η:V×V→[0,1] satisfying η(x,y)≤min⁡{ξ(x),ξ(y)}.

Definition 2.2 [40] Degree of a vertex *v* is denoted by $d_{G}(v)$ or simply d(v) and defined as $d(v)=\sum_{x\in V}\eta (xv)$.

Here, maximum degree of *G* is denoted by Δ(G) or Δ and is defined as $\Delta =\vee_{v\in V}d(v)$. Also, minimum degree of *G* is denoted by δ(G) or *δ* and is defined as $\delta =\wedge_{v\in V}d(v)$. The total degree of *G* is sum of the degrees of all vertices and denoted by T(G) or simply *T*.

Throughout this article, the FG, $G_{1}=(V_{1},E_{1})$ has $n_{1}$-vertices, $m_{1}$-edges and $G_{2}=(V_{2},E_{2})$ has $n_{2}$-vertices, $m_{2}$-edges and $\Delta_{1}=\Delta (G_{1}),\Delta_{2}=\Delta (G_{2}),\delta_{1}=\delta (G_{1}),\delta_{2}=\delta (G_{2})$.

Now, Cartesian product, composition, join, union, direct product, strong product and semi-strong product of two FGs are defined below:

Definition 2.3 [40] The Cartesian product of $G_{1}$ and $G_{2}$ is a FG $G_{1}\times G_{2}=(V,E)$, where $V=V_{1}\times V_{2}$, $\left(u,v),(u_{1},v_{1}),(u_{2},v_{2})\in V,\xi (u,v)=\wedge \{\xi_{1}(u),\xi_{2}(v)\right\}$ and

$$\eta ((u_{1},v_{1}),(u_{2},v_{2}))=\left\{ \begin{array}{c} \wedge \{\xi_{1}(u_{1}),\eta_{2}(v_{1},v_{2})\}\text{ if }u_{1}=u_{2},v_{1}v_{2}\in E_{2}\\ \wedge \{\xi_{2}(v_{1}),\eta_{1}(u_{1},u_{2})\}\text{ if }u_{1}u_{2}\in E_{1},v_{1}=v_{2}\\ 0\text{ otherwise.} \end{array}\right.$$

Definition 2.4 [40] Let $G_{1}=(V_{1},E_{1}),G_{2}=(V_{2},E_{2})$ be two FGs. Then the composition of $G_{1}$ and $G_{2}$ is a FG $G_{1}[G_{2}]=(V,E)$, where $V=V_{1}\times V_{2}$, $\left(u,v),(u_{1},v_{1}\right)$, $\left(u_{2},v_{2})\in V,\xi (u,v)=\wedge \{\xi_{1}(u),\xi_{2}(v)\right\}$ and

$$\eta ((u_{1},v_{1}),(u_{2},v_{2}))=\left\{ \begin{array}{c} \wedge \{\xi_{1}(u_{1}),\eta_{2}(v_{1},v_{2})\}\text{ if }u_{1}=u_{2}\text{ and }v_{1}v_{2}\in E_{2}\\ \wedge \{\xi_{2}(v_{1}),\xi_{2}(v_{2}),\eta_{1}(u_{1},u_{2})\}\text{ if }u_{1}u_{2}\in E_{1}\\ 0\text{ otherwise.} \end{array}\right.$$

Definition 2.5 [40] Let $G_{1}=(V_{1},E_{1}),G_{2}=(V_{2},E_{2})$ be two FGs. Then the join of $G_{1}$ and $G_{2}$ is a FG $G_{1}+G_{2}=(V,E)$, where $V=V_{1}\cup V_{2}$, $u,v,u_{1},v_{1}\in V$,

$$\xi (u)=\left\{ \begin{array}{c} \xi_{1}(u),\text{ if }u\in V_{1}\\ \xi_{2}(u),\text{ if }u\in V_{2} \end{array}\right.$$

and

$$\eta (u,v)=\left\{ \begin{array}{c} \wedge \{\xi_{1}(u),\xi_{2}(v)\}\text{ if }u\in V_{1}\text{ and }v\in V_{2}\\ \eta_{1}(u,v)\text{ if }uv\in E_{1}\\ \eta_{2}(u,v)\text{ if }uv\in E_{2}\\ 0\text{ otherwise.} \end{array}\right.$$

Definition 2.6 [40] Let $G_{1}=(V_{1},E_{1}),G_{2}=(V_{2},E_{2})$ be two FGs. Then the union of $G_{1}$ and $G_{2}$ is a FG $G_{1}\cup G_{2}=(V,E)$, where $V=V_{1}\cup V_{2}$, u,v∈V,

$$\xi (u)=\left\{ \begin{array}{c} \xi_{1}(u),\text{ if }u\in V_{1}\setminus V_{2}\\ \xi_{2}(u),\text{ if }u\in V_{2}\setminus V_{1}\\ \vee \{\xi_{1}(u),\xi_{2}(u)\},\text{ if }u\in V_{1}\cap V_{2} \end{array}\right.$$

and

$$\eta (u,v)=\left\{ \begin{array}{c} \eta_{1}(u,v)\text{ if }uv\in E_{1}\setminus E_{2}\\ \eta_{2}(u,v)\text{ if }uv\in E_{2}\setminus E_{1}\\ \vee \{\xi_{1}(u,v),\xi_{2}(u,v)\}\text{ if }(u,v)\in E_{1}\cap E_{2}\\ 0\text{ otherwise.} \end{array}\right.$$

Definition 2.7 [40] Let $G_{1}=(V_{1},E_{1}),G_{2}=(V_{2},E_{2})$ be two FGs. Suppose, $V_{1}\cap V_{2}=\phi$, then the direct product of $G_{1}$ and $G_{2}$ is a FG $G_{1}\prod G_{2}=(V,E)$, where $V=V_{1}\times V_{2}$, $E=\{((u_{1},v_{1})(u_{2},v_{2})):u_{1}u_{2}\in E_{1}\text{ and }v_{1}v_{2}\in E_{2}\}$, $\xi (u,v)=\xi_{1}(u)\wedge \xi_{2}(v)$ and $\eta ((u_{1},v_{1})(u_{2},v_{2}))=\eta_{1}(u_{1}u_{2})\wedge \eta_{2}(v_{1}v_{2})$.

Definition 2.8 [40] Let $G_{1}=(V_{1},E_{1}),G_{2}=(V_{2},E_{2})$ be two FGs. Suppose, $V_{1}\cap V_{2}=\phi$, then the semi-strong product of $G_{1}$ and $G_{2}$ is a FG $G_{1}•G_{2}=(V,E)$, where $V=V_{1}\times V_{2}$, $E=\{((u_{1},v_{1})(u_{2},v_{2})):u_{1}u_{2}\in E_{1}\text{ and }v_{1}v_{2}\in E_{2}\}\cup \{((u,v_{1})(u,v_{2})):u\in V_{1},v_{1}v_{2}\in E_{2}\}$, $\xi (u,v)=\xi_{1}(u)\wedge \xi_{2}(v)$ and

$$\eta ((u_{1},v_{1})(u_{2},v_{2}))=\left\{ \begin{array}{c} \eta_{1}(u_{1}u_{2})\wedge \eta_{2}(v_{1}v_{2})\text{ if }u_{1}u_{2}\in E_{1}\text{ and }v_{1}v_{2}\in E_{2}\\ \xi_{1}(u)\wedge \eta_{2}(v_{1}v_{2})\text{ if }u_{1}=u_{2}=u\in V_{1}\text{ and }v_{1}v_{2}\in E_{2} \end{array}\right.$$

Definition 2.9 [40] Let $G_{1}=(V_{1},E_{1}),G_{2}=(V_{2},E_{2})$ be two FGs. Suppose, $V_{1}\cap V_{2}=\phi$, then the strong product of $G_{1}$ and $G_{2}$ is a FG $G_{1}⊗G_{2}=(V,E)$, where $V=V_{1}\times V_{2}$, $E=\{((u_{1},v_{1})(u_{2},v_{2})):u_{1}u_{2}\in E_{1}\text{ and }v_{1}v_{2}\in E_{2}\}\cup \{((u,v_{1})(u,v_{2})):u\in V_{1},v_{1}v_{2}\in E_{2}\}$, $\xi (u,v)=\xi_{1}(u)\wedge \xi_{2}(v)$ and

$$\eta ((u_{1},v_{1})(u_{2},v_{2}))=\left\{ \begin{array}{c} \eta_{1}(u_{1}u_{2})\wedge \eta_{2}(v_{1}v_{2})\text{ if }u_{1}u_{2}\in E_{1}\text{ and }v_{1}v_{2}\in E_{2}\\ \xi_{1}(u)\wedge \eta_{2}(v_{1}v_{2})\text{ if }u_{1}=u_{2}=u\in V_{1}\text{ and }v_{1}v_{2}\in E_{2}\\ \xi_{2}(v)\wedge \eta_{1}(u_{1}u_{2})\text{ if }u_{1}u_{2}\in E_{1}\text{ and }v_{1}=v_{2}=v\in V_{2} \end{array}\right.$$

In 2021, Islam and Pal [23] introduced the first ZI for FGs as:

Definition 2.10 [23] Let *G* be a connected FG. Then first ZI of *G* is defined by:

$$FZI_{1}(G)=\sum_{v\in V}{\left[\xi (v)deg(v)\right]}^{2}.$$

Theorem 2.1 *[23]* *Suppose G be a FG having n vertices and m edges. Then,*

*(i)* $FZI_{1}(G)\leq 4n^{2}m^{2}$ *, (ii)* $FZI_{1}(G)\leq n^{2}T^{2}$ *and (iii)* $FZI_{1}(G)\leq n\Delta^{2}$ *.*

Definition 2.11 [23] Let *G* be a connected FG. Then second-ZI of *G* is defined by:

$$FZI_{2}(G)=\sum_{uv\in E}[\xi (u)deg(u)\xi (v)deg(v)].$$

## 3 Hyper-Zagreb index for fuzzy graph

TIs have a vital role in chemical graph theory. ZIs are such topological descriptors which are degree-based TIs, applied to evaluate *π*-electron energy of some special type of chemical compound and established by Gutman and Trinajstic [14] in 1972. Now, hyper-ZI is defined as:

Definition 3.1 Let G=(V,E) be a connected crisp graph. Then hyper-ZI of the graph *G* is denoted by HZI(G) and is defined by:

$$HZI(G)=\sum_{uv\in E}{\left[deg(u)+deg(v)\right]}^{2}.$$

In this section, hyper-ZI for a FG is defined and discussed by an example. Then, hyper-ZI is studied for various FGs like path, cycle, star, complete-FG, fuzzy subgraph, partial fuzzy subgraph, etc.

Definition 3.2 Let G=(V,E) be a connected FG. Then hyper-ZI of the FG *G* is denoted by FHZI(G) and is defined by:

$$FHZI(G)=\sum_{uv\in E}{\left[\xi (u)deg(u)+\xi (v)deg(v)\right]}^{2}.$$

**Figure 1:**
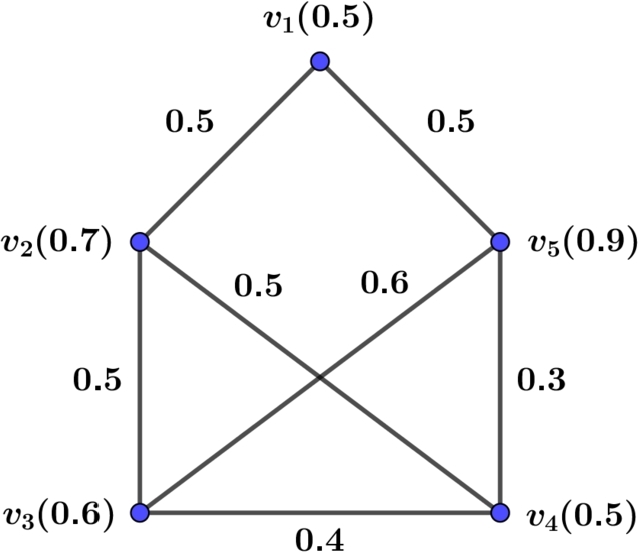
A FG *G* with *FHZI*(*G*)=20.7427.

Example 3.1 Suppose G=(V,E) be a FG shown in Fig. 1 with $V=\{v_{1},v_{2},v_{3},v_{4},v_{5}\}$ and $E=\{v_{1}v_{2},v_{1}v_{5},v_{2}v_{3},v_{2}v_{4},v_{3}v_{4},v_{3}v_{5},v_{4}v_{5}\}$ where $\xi (v_{1})=0.5,\xi (v_{2})=0.7,\xi (v_{3})=0.6,\xi (v_{4})=0.5,\xi (v_{5})=0.9,\eta (v_{1}v_{2})=0.5,\eta (v_{1}v_{5})=0.0.5,\eta (v_{2}v_{3})=0.5,\eta (v_{2}v_{4})=0.5,\eta (v_{3}v_{4})=0.4,\eta (v_{3}v_{5})=0.6,\eta (v_{4}v_{5})=0.3$. Then degree of the vertices is:

$$deg(v_{1})=\sum_{vv_{1}\in E}\eta (vv_{1})=\eta (v_{1}v_{2})+\eta (v_{1}v_{5})=0.5+0.5=1.0,deg(v_{2})=\sum_{vv_{2}\in E}\eta (vv_{2})=\eta (v_{1}v_{2})+\eta (v_{2}v_{3})+\eta (v_{2}v_{4})=0.5+0.5+0.5=1.5,deg(v_{3})=\sum_{vv_{3}\in E}\eta (vv_{3})=\eta (v_{2}v_{3})+\eta (v_{3}v_{4})+\eta (v_{3}v_{5})=0.5+0.4+0.6=1.5,deg(v_{4})=\sum_{vv_{4}\in E}\eta (vv_{4})=\eta (v_{2}v_{4})+\eta (v_{3}v_{4})+\eta (v_{4}v_{5})=0.5+0.4+0.3=1.2\;deg(v_{5})=\sum_{vv_{5}\in E}\eta (vv_{5})=\eta (v_{1}v_{5})+\eta (v_{3}v_{5})+\eta (v_{4}v_{5})=0.5+0.6+0.3=1.4.$$

Then,

$$FHZI(G)=\sum_{uv\in E}{\left[\xi (u)deg(u)+\xi (v)deg(v)\right]}^{2}={\left(0.5\times 1.0+0.7\times 1.5\right)}^{2}+{\left(0.5\times 1.0+0.5\times 1.5\right)}^{2}+{\left(0.7\times 1.5+0.6\times 1.5\right)}^{2}+{\left(0.7\times 1.5+0.5\times 1.2\right)}^{2}+{\left(0.6\times 1.5+0.5\times 1.2\right)}^{2}+{\left(0.6\times 1.5+0.9\times 1.4\right)}^{2}+{\left(0.5\times 1.2+0.9\times 1.4\right)}^{2}=20.7427.$$

Now, this index is studied for a path.

Theorem 3.1 *Let P be a path. Then,* $FHZI(P)\leq 2(4|V(P)|-7)+2FZI_{2}(P)$ *.*

Proof From [20], [21], [23], we get,

$$FHZI(P)=\sum_{uv\in E}{\left[\xi (u)deg(u)+\xi (v)deg(v)\right]}^{2}=\sum_{uv\in E}[\xi^{2}(u)deg^{2}(u)+\xi^{2}(v)deg^{2}(v)]+2\sum_{uv\in E}[\xi (u)deg(u)\xi (v)deg(v)]=\xi^{2}(u_{1})deg^{2}(u_{1})+\xi^{2}(u_{n})deg^{2}(u_{n})+2\sum_{i=2}^{n-1}[\xi^{2}(u_{i})deg^{2}(u_{i})]+2FZI_{2}(P)\leq 2+2\sum_{i=2}^{n-1}2^{2}+2FZI_{2}(P)=2(4n-7)+2FZI_{2}(P).$$

 □

Theorem 3.2 *Let C be a cycle. Then,* $FHZI(C)\leq 8|V(C)|+2FZI_{2}(C)$ *.*

Proof From [20], [21], [23], we get,

$$FHZI(C)=\sum_{uv\in E}{\left[\xi (u)deg(u)+\xi (v)deg(v)\right]}^{2}=\sum_{uv\in E}[\xi^{2}(u)deg^{2}(u)+\xi^{2}(v)deg^{2}(v)]+2\sum_{uv\in E}[\xi (u)deg(u)\xi (v)deg(v)]=2\sum_{i=1}^{n}{\left[\xi (u_{i})deg(u_{i})\right]}^{2}+2FZI_{2}(C)\leq 2\sum_{i=1}^{n}2^{2}+2FZI_{2}(C)=8n+2FZI_{2}(C).$$

 □

Theorem 3.3 *Suppose, G be a saturated fuzzy cycle with each α-strong edges has membership value α and each β-strong edges has membership value β. Then,* $FHZI(G)\leq 4|V(G)|{\left(\alpha +\beta \right)}^{2}$ *.*

Proof As *G* is a saturated fuzzy cycle with each *α*-strong edges has membership value *α* and each *β*-strong edges have membership value *β*, the degree of each vertex of *G* is α+β. Hence,

$$FHZI(G)=\sum_{uv\in E}{\left[\xi (u)deg(u)+\xi (v)deg(v)\right]}^{2}=\sum_{uv\in E}{\left[\xi (u)(\alpha +\beta )+\xi (v)(\alpha +\beta )\right]}^{2}\leq 4|V(G)|{\left(\alpha +\beta \right)}^{2}.$$

Now, this index is studied for a star. □

Theorem 3.4 *Let S be a star. Then,* $FHZI(S)\leq [|V(S)|-1][{\left(|V(S)|-1\right)}^{2}+1]+2FZI_{2}(S)$ *.*

Proof From [20], [21], [23], we get,

$$FHZI(S)=\sum_{uv\in E}{\left[\xi (u)deg(u)+\xi (v)deg(v)\right]}^{2}=\sum_{uv\in E}[\xi^{2}(u)deg^{2}(u)+\xi^{2}(v)deg^{2}(v)]+2\sum_{uv\in E}[\xi (u)deg(u)\xi (v)deg(v)]=n{\left[\xi (u_{0})deg(u_{0})\right]}^{2}+\sum_{i=1}^{n}{\left[\xi (u_{i})deg(u_{i})\right]}^{2}+2FZI_{2}(S)\leq 2FZI_{2}(S)+n^{3}+\sum_{i=1}^{n}1=n(n^{2}+1)+2FZI_{2}(S).$$

 □

Theorem 3.5 *Suppose a connected partial fuzzy subgraph H of a FG G. Then,* FHZI(H)≤FHZI(G) *.*

Proof From [20], [21], [23], we get,

$$FHZI(H)=\sum_{uv\in E(H)}{\left[\xi_{H}(u)deg_{H}(u)+\xi_{H}(v)deg_{H}(v)\right]}^{2}\leq \sum_{uv\in E(H)}{\left[\xi_{G}(u)deg_{G}(u)+\xi_{G}(v)deg_{G}(v)\right]}^{2}\leq \sum_{uv\in E(G)}{\left[\xi_{G}(u)deg_{G}(u)+\xi_{G}(v)deg_{G}(v)\right]}^{2}=FHZI(G).$$

Let *G* be any connected FG. Now C(G) is considered as a complete FG with vertex set is V(G) and membership value of each vertex in C(G) is the membership value of that vertex for the FG *G*. Then clearly, *G* is a connected partial fuzzy subgraph of the FG C(G). □

Corollary 3.1 *Let G be any connected FG. Then,* FHZI(G)≤FHZI(C(G)) *.*

Theorem 3.6 *Let G be a complete FG. Then,* $FHZI(G)\leq |V(G)|{\left[|V(G)|-1\right]}^{3}+2FZI_{2}(G)$ *.*

Proof From [20], [21], [23], we get,

$$FHZI(G)=\sum_{uv\in E}{\left[\xi (u)deg(u)+\xi (v)deg(v)\right]}^{2}=\sum_{uv\in E}[\xi^{2}(u)deg^{2}(u)+\xi^{2}(v)deg^{2}(v)]+2\sum_{uv\in E}[\xi (u)deg(u)\xi (v)deg(v)]=(n-1)\sum_{i=1}^{n}{\left[\xi (u_{i})deg(u_{i})\right]}^{2}+2FZI_{2}(G)=(n-1)\sum_{i=1}^{n}{\left[\xi (u_{i})\{\sum_{j=1}^{i-1}\xi (u_{j})+(n-i)\xi (u_{i})\}\right]}^{2}+2FZI_{2}(G)\leq (n-1)\sum_{i=1}^{n}{\left[\sum_{j=1}^{i-1}1+(n-i)\right]}^{2}+2FZI_{2}(G)=(n-1)\sum_{i=1}^{n}{\left[n-1\right]}^{2}+2FZI_{2}(G)=n{\left(n-1\right)}^{3}+2FZI_{2}(G).$$

 □

Theorem 3.7 *Let* $G_{1}$ *and* $G_{2}$ *be two isomorphic connected FGs. Then,* $FHZI(G_{1})=FHZI(G_{2})$ *.*

Proof As $G_{1}$ and $G_{2}$ be two isomorphic connected FG there exist a bijective map $\psi :V(G_{1})\to V(G_{2})$ such that, $\xi_{G_{1}}(v)=\xi_{G_{2}}(\psi (v))$ and $\eta_{G_{1}}(uv)=\eta_{G_{2}}(\psi (u)\psi (v)),\forall u,v\in V(G_{1})$ and u≠v. Then,

$$deg_{G_{1}}(v)=\sum_{u\in VG_{1}}\eta_{G_{1}}(uv)=\sum_{\psi (u)\in VG_{2}}\eta_{G_{2}}(\psi (u)\psi (v))=deg_{G_{2}}(\psi (v)).$$

Therefore,

$$FHZI(G_{1})=\sum_{uv\in E(G_{1})}{\left[\xi_{G_{1}}(u)deg_{G_{1}}(u)+\xi_{G_{1}}(v)deg_{G_{1}}(v)\right]}^{2}=\sum_{\psi (u)\psi (v)\in E(G_{2})}{\left[\xi_{G_{2}}(\psi (u))deg_{G_{2}}(\psi (u))+\xi_{G_{2}}(\psi (v))deg_{G_{2}}(\psi (v))\right]}^{2}=FHZI(G_{2}).$$

Here, a relation between hyper-ZI and second ZI has been established. □

Theorem 3.8 *Suppose, G be a FG. Then,* $FHZI(G)\geq 4FZI_{2}(G)$ *.*

Proof As *G* is a FG, then,

$$FHZI(G)=\sum_{uv\in E}{\left[\xi (u)deg(u)+\xi (v)deg(v)\right]}^{2}=\sum_{uv\in E}[\xi^{2}(u)deg^{2}(u)+\xi^{2}(v)deg^{2}(v)]+2\sum_{uv\in E}\xi (u)\xi (v)deg(u)deg(v)\geq 2\sum_{uv\in E}\xi (u)\xi (v)deg(u)deg(v)+2\sum_{uv\in E}\xi (u)\xi (v)deg(u)deg(v)=4FZI_{2}(G).$$

 □

## 4 Bounds on hyper-Zagreb index for fuzzy graphs during operations

In this section, some bounds are established related to hyper-ZI for FGs during FG operations.

Theorem 4.1 $FHZI(G_{1}\times G_{2})\leq 2[m_{1}FZI_{1}(G_{2})+m_{2}FZI_{2}(G_{1})+FZI_{2}(G_{1}\times G_{2})+(n_{1}m_{2}\Delta_{2}+n_{2}m_{1}\Delta_{1})(\Delta_{1}+\Delta_{2})]$ *.*

Proof As $G_{1}\times G_{2}$ is CP of $G_{1}$ and $G_{2}$, then for $\left(u,v),(u_{1},v_{1})\in V,\xi (u,v)=\wedge \{\xi_{1}(u),\xi_{2}(v)\right\}$ and

$$\eta ((u,v)(u_{1},v_{1}))=\left\{ \begin{array}{c} \wedge \{\xi_{1}(u),\eta_{2}(vv_{1})\}\;\text{ if }u=u_{1},vv_{1}\in E_{2}\\ \wedge \{\xi_{2}(v),\eta_{1}(uu_{1})\}\;\text{ if }uu_{1}\in E_{1},v=v_{1}\\ 0\;\text{otherwise.} \end{array}\right.$$

$$\text{Then }deg_{G_{1}\times G_{2}}(u,v)=\sum_{v_{i}v\in E_{2}}\eta \{(u,v)(u,v_{i})\}+\sum_{u_{i}u\in E_{1}}\eta \{(u,v)(u_{i},v)\}=\sum_{v_{i}v\in E_{2}}\wedge \{\xi_{1}(u),\eta_{2}(vv_{i})\}+\sum_{u_{i}u\in E_{1}}\wedge \{\xi_{2}(v),\eta_{1}(uu_{i})\}\leq \sum_{v_{i}v\in E_{2}}\eta_{2}(vv_{i})+\sum_{u_{i}u\in E_{1}}\eta_{1}(uu_{i})=deg_{G_{1}}(u)+deg_{G_{2}}(v).$$

$$\text{Now }FHZI(G_{1}\times G_{2})=\sum_{(u_{1},v_{1})(u_{2},v_{2})\in E}{\left[\xi (u_{1},v_{1})deg_{G_{1}\times G_{2}}(u_{1},v_{1})+\xi (u_{2},v_{2})deg_{G_{1}\times G_{2}}(u_{2},v_{2})\right]}^{2}=\sum_{(u_{1},v_{1})(u_{2},v_{2})\in E}[\xi^{2}(u_{1},v_{1})deg_{G_{1}\times G_{2}}^{2}(u_{1},v_{1})+\xi^{2}(u_{2},v_{2})deg_{G_{1}\times G_{2}}^{2}(u_{2},v_{2})]+2\sum_{(u_{1},v_{1})(u_{2},v_{2})\in E}[\xi (u_{1},v_{1})\xi (u_{2},v_{2})deg_{G_{1}\times G_{2}}(u_{1},v_{1})deg_{G_{1}\times G_{2}}(u_{2},v_{2})]=\underset{K_{1}}{\underset{︸}{\sum_{u\in V_{1}v_{1}v_{2}E_{2}}[\xi^{2}(u,v_{1})deg_{G_{1}\times G_{2}}^{2}(u,v_{1})+\xi^{2}(u,v_{2})deg_{G_{1}\times G_{2}}^{2}(u,v_{2})]}}+\underset{K_{2}}{\underset{︸}{\sum_{v\in V_{2}u_{1}u_{2}E_{1}}[\xi^{2}(u_{1},v)deg_{G_{1}\times G_{2}}^{2}(u_{1},v)+\xi^{2}(u_{2},v)deg_{G_{1}\times G_{2}}^{2}(u_{2},v)]}}+2FZI_{2}(G_{1}\times G_{2}).$$

$$\text{Now }K_{1}=\sum_{u\in V_{1}v_{1}v_{2}E_{2}}[\xi^{2}(u,v_{1})deg_{G_{1}\times G_{2}}^{2}(u,v_{1})+\xi^{2}(u,v_{2})deg_{G_{1}\times G_{2}}^{2}(u,v_{2})]\leq \sum_{u\in V_{1}v_{1}v_{2}E_{2}}[\xi^{2}(u)deg_{G_{1}}^{2}(u)+\xi (u)\xi (v_{1})deg_{G_{1}}(u)deg_{G_{2}}(v_{1})+\xi^{2}(v_{1})deg_{G_{2}}^{2}(v_{1})+\xi^{2}(u)deg_{G_{1}}^{2}(u)+\xi (u)\xi (v_{2})deg_{G_{1}}(u)deg_{G_{2}}(v_{2})+\xi^{2}(v_{2})deg_{G_{2}}^{2}(v_{2})]\leq 2m_{2}\sum_{u\in V_{1}}{\left[\xi (u)deg_{G_{1}}(u)\right]}^{2}+\sum_{u\in V_{1}}\sum_{v_{1}v_{2}E_{2}}[\xi^{2}(v_{1})deg_{G_{2}}^{2}(v_{1})+\xi^{2}(v_{2})deg_{G_{2}}^{2}(v_{2})]+\sum_{u\in V_{1}}\xi (u)deg_{G_{1}}(u)\sum_{v_{1}v_{2}E_{2}}[\xi (v_{1})deg_{G_{2}}(v_{1})+\xi (v_{2})deg_{G_{2}}(v_{2})]\leq 2m_{2}FZI_{1}(G_{1})+2n_{1}m_{2}\Delta_{1}\Delta_{2}+2n_{1}m_{2}\Delta_{2}^{2}=2m_{2}FZI_{1}(G_{1})+2n_{1}m_{2}\Delta_{2}(\Delta_{1}+\Delta_{2}).$$

$$\text{Simillarly }K_{2}\leq 2m_{1}FZI_{1}(G_{2})+2n_{2}m_{1}\Delta_{1}(\Delta_{1}+\Delta_{2}).∴FHZI(G_{1}\times G_{2})\leq 2[m_{1}FZI_{1}(G_{2})+m_{2}FZI_{1}(G_{1})+FZI_{2}(G_{1}\times G_{2})+(n_{1}m_{2}\Delta_{2}+n_{2}m_{1}\Delta_{1})(\Delta_{1}+\Delta_{2})].$$

 □

Theorem 4.2 $FHZI(G_{1}[G_{2}])\leq 2[m_{2}n_{2}^{2}FZI_{1}(G_{1})+m_{1}FZI_{1}(G_{2})+FZI_{2}(G_{1}[G_{2}])+m_{1}n_{2}^{4}\Delta_{1}^{2}+n_{1}m_{2}\Delta_{2}^{2}+2n_{2}(m_{1}n_{2}^{2}+m_{2}n_{1})\Delta_{1}\Delta_{2}]$ *.*

Proof As $G_{1}[G_{2}]$ is composition graph of $G_{1}$ and $G_{2}$, then for $\left(u,v),(u_{1},v_{1})\in V,\xi (u,v)=\wedge \{\xi_{1}(u),\xi_{2}(v)\right\}$ and

$$\eta ((u,v)(u_{1},v_{1}))=\left\{ \begin{array}{c} \wedge \{\xi_{1}(u),\eta_{2}(vv_{1})\}\;\text{ if }u=u_{1},vv_{1}\in E_{2}\\ \wedge \{\xi_{2}(v),\xi_{2}(v_{1}),\eta_{1}(uu_{1})\}\;\text{ if }uu_{1}\in E_{1}\\ 0\;\text{ otherwise.} \end{array}\right.$$

$$\text{Then }deg_{G_{1}[G_{2}]}(u,v)=\sum_{v_{i}v\in E_{2}}\eta \{(u,v)(u,v_{i})\}+\sum_{u_{i}u\in E_{1},v_{j}\in V_{2}}\eta \{(u,v)(u_{i},v_{j})\}=\sum_{v_{i}v\in E_{2}}\wedge \{\xi_{1}(u),\eta_{2}(vv_{i})\}+\sum_{u_{i}u\in E_{1},v_{j}\in V_{2}}\wedge \{\xi_{2}(v),\xi_{2}(v_{j})\eta_{1}(uu_{i})\}\leq \sum_{v_{i}v\in E_{2}}\eta_{2}(vv_{i})+\sum_{u_{i}u\in E_{1},v_{j}\in V_{2}}\eta_{1}(uu_{i})=n_{2}deg_{G_{1}}(u)+deg_{G_{2}}(v).$$

$$\text{Now, }FHZI(G_{1}[G_{2}])=\sum_{(u_{1},v_{1})(u_{2},v_{2})\in E}{\left[\xi (u_{1},v_{1})deg_{G_{1}[G_{2}]}(u_{1},v_{1})+\xi (u_{2},v_{2})deg_{G_{1}[G_{2}]}(u_{2},v_{2})\right]}^{2}=\sum_{(u_{1},v_{1})(u_{2},v_{2})\in E}[\xi^{2}(u_{1},v_{1})deg_{G_{1}[G_{2}]}^{2}(u_{1},v_{1})+\xi^{2}(u_{2},v_{2})deg_{G_{1}[G_{2}]}^{2}(u_{2},v_{2})]+2\sum_{(u_{1},v_{1})(u_{2},v_{2})\in E}[\xi (u_{1},v_{1})\xi (u_{2},v_{2})deg_{G_{1}[G_{2}]}(u_{1},v_{1})deg_{G_{1}[G_{2}]}(u_{2},v_{2})]=\underset{K_{1}}{\underset{︸}{\sum_{u\in V_{1},v_{1}v_{2}\in E_{2}}[\xi^{2}(u,v_{1})deg_{G_{1}[G_{2}]}^{2}(u,v_{1})+\xi^{2}(u,v_{2})deg_{G_{1}[G_{2}]}^{2}(u,v_{2})]}}+\underset{K_{2}}{\underset{︸}{\sum_{u_{1}u_{2}\in E_{1},v_{1}v_{2}\in V_{2}}[\xi^{2}(u_{1},v_{1})deg_{G_{1}[G_{2}]}^{2}(u_{1},v_{1})+\xi^{2}(u_{2},v_{2})deg_{G_{1}[G_{2}]}^{2}(u_{2},v_{2})]}}+2FZI_{2}(G_{1}[G_{2}]).$$

$$\text{Now }K_{1}=\sum_{u\in V_{1},v_{1}v_{2}\in E_{2}}[\xi^{2}(u,v_{1})deg_{G_{1}[G_{2}]}^{2}(u,v_{1})+\xi^{2}(u,v_{2})deg_{G_{1}[G_{2}]}^{2}(u,v_{2})]\leq \sum_{u\in V_{1},v_{1}v_{2}\in E_{2}}[{\left\{n_{2}\xi_{1}(u)deg_{G_{1}}(u)+\xi_{2}(v_{1})deg_{G_{2}}(v_{1})\right\}}^{2}+{\left\{n_{2}\xi_{1}(u)deg_{G_{1}}(u)+\xi_{2}(v_{2})deg_{G_{2}}(v_{2})\right\}}^{2}]=2n_{2}^{2}\sum_{u\in V_{1},v_{1}v_{2}\in E_{2}}\xi_{1}^{2}(u)deg_{G_{1}}^{2}(u)+\sum_{u\in V_{1},v_{1}v_{2}\in E_{2}}[\xi_{2}^{2}(v_{1})deg_{G_{2}}^{2}(v_{1})+\xi_{2}^{2}(v_{2})deg_{G_{2}}^{2}(v_{2})]+2n_{2}\sum_{u\in V_{1}}\xi_{1}(u)deg_{G_{1}}(u)\sum_{v_{1}v_{2}\in E_{2}}[\xi_{2}(v_{1})deg_{G_{2}}(v_{1})+\xi_{2}(v_{2})deg_{G_{2}}(v_{2})]\leq 2m_{2}n_{2}^{2}FZI_{1}(G_{1})+2n_{1}m_{2}\Delta_{2}^{2}+4n_{1}n_{2}m_{2}\Delta_{1}\Delta_{2}.$$

$$\text{Again }K_{2}=\sum_{u_{1}u_{2}\in E_{1},v_{1}v_{2}\in V_{2}}[\xi^{2}(u_{1},v_{1})deg_{G_{1}[G_{2}]}^{2}(u_{1},v_{1})+\xi^{2}(u_{2},v_{2})deg_{G_{1}[G_{2}]}^{2}(u_{2},v_{2})]\leq \sum_{u_{1}u_{2}\in E_{1},v_{1},v_{2}\in V_{2}}[{\left\{n_{2}\xi_{1}(u_{1})deg_{G_{1}}(u_{1})+\xi_{2}(v_{1})deg_{G_{2}}(v_{1})\right\}}^{2}+{\left\{n_{2}\xi_{1}(u_{2})deg_{G_{1}}(u_{2})+\xi_{2}(v_{2})deg_{G_{2}}(v_{2})\right\}}^{2}]=n_{2}^{2}\sum_{u_{1}u_{2}\in E_{1},v_{1},v_{2}\in V_{2}}[\xi_{1}^{2}(u_{1})deg_{G_{1}}^{2}(u_{1})+\xi_{1}^{2}(u_{2})deg_{G_{1}}^{2}(u_{2})]+\sum_{u_{1}u_{2}\in E_{1},v_{1},v_{2}\in V_{2}}[\xi_{2}^{2}(v_{1})deg_{G_{2}}^{2}(v_{1})+\xi_{2}^{2}(v_{2})deg_{G_{2}}^{2}(v_{2})]+2n_{2}\sum_{u_{1}u_{2}\in E_{1},v_{1},v_{2}\in V_{2}}[\xi_{1}(u_{1})\xi_{2}(v_{1})deg_{G_{2}}(u_{1})deg_{G_{2}}(v_{1})+\xi_{1}(u_{2})\xi_{2}(v_{2})deg_{G_{2}}(u_{2})deg_{G_{2}}(v_{2})]\leq 2m_{1}FZI_{1}(G_{2})+2m_{1}n_{2}^{4}\Delta_{1}^{2}+4m_{1}n_{2}^{3}\Delta_{1}\Delta_{2}.$$

$$∴FHZI(G_{1}[G_{2}])\leq 2[m_{2}n_{2}^{2}FZI_{1}(G_{1})+m_{1}FZI_{1}(G_{2})+FZI_{2}(G_{1}[G_{2}])+m_{1}n_{2}^{4}\Delta_{1}^{2}+n_{1}m_{2}\Delta_{2}^{2}+2n_{2}(m_{1}n_{2}^{2}+m_{2}n_{1})\Delta_{1}\Delta_{2}].$$

 □

Theorem 4.3 $n_{2}FZI_{1}(G_{1})+n_{1}FZI_{1}(G_{2})+2FZI_{2}(G_{1})+2FZI_{2}(G_{2})+FZI_{2}(G_{1}+G_{2})\leq FHZI(G_{1}+G_{2})\leq n_{2}FZI_{1}(G_{1})+n_{1}FZI_{1}(G_{2})+FZI_{2}(G_{1}+G_{2})+n_{1}n_{2}(n_{1}^{2}+n_{2}^{2})+2n_{1}n_{2}(n_{2}\Delta_{1}+n_{1}\Delta_{2})+2m_{1}{\left(n_{2}+\Delta_{1}\right)}^{2}+2m_{2}{\left(n_{1}+\Delta_{2}\right)}^{2}$ *.*

Proof As $G_{1}+G_{2}$ is join of $G_{1}$ and $G_{2}$, then for u,v∈V,

$$\xi (u)=\left\{ \begin{array}{c} \xi_{1}(u),\;\text{if }u\in V_{1}\\ \xi_{2}(u),\;\text{otherwise} \end{array}\right.$$

$$\text{and }\eta (uv)=\left\{ \begin{array}{c} \wedge \{\xi_{1}(u),\xi_{2}(v)\}\;\text{ if }u\in V_{1},v\in V_{2}\\ \eta_{1}(uv)\;\text{ if }uv\in E_{1}\\ \eta_{2}(uv)\;\text{ if }uv\in E_{2}\\ 0\;\text{otherwise.} \end{array}\right.$$

Then,

$$deg_{G_{1}+G_{2}}(u)=\left\{ \begin{array}{c} deg_{G_{1}}(u)+\sum_{v\in V_{2}}\wedge \{\xi_{1}(u),\xi_{2}(v)\},\;u\in V_{1}\\ deg_{G_{2}}(u)+\sum_{v\in V_{1}}\wedge \{\xi_{2}(u),\xi_{1}(v)\},\;u\in V_{2} \end{array}\right.\geq \left\{ \begin{array}{c} deg_{G_{1}}(u),\;u\in V_{1}\\ deg_{G_{2}}(u),\;u\in V_{2}. \end{array}\right.$$

Also, the followings are hold:

$$deg_{G_{1}+G_{2}}(u)\leq \left\{ \begin{array}{c} deg_{G_{1}}(u)+\sum_{v\in V_{2}}\xi_{1}(u),\;u\in V_{1}\\ deg_{G_{2}}(u)+\sum_{v\in V_{1}}\xi_{2}(u),\;u\in V_{2} \end{array}\right.\leq \left\{ \begin{array}{c} deg_{G_{1}}(u)+n_{2}\xi_{1}(u),\;u\in V_{1}\\ deg_{G_{2}}(u)+n_{1}\xi_{2}(u),\;u\in V_{2} \end{array}\right.$$

$$\text{Now, }FHZI(G_{1}+G_{2})=\sum_{uv\in E}{\left[\xi (u)deg_{G_{1}+G_{2}}(u)+\xi (v)deg_{G_{1}+G_{2}}(v)\right]}^{2}=\sum_{uv\in E}[\xi^{2}(u)deg_{G_{1}+G_{2}}^{2}(u)+\xi^{2}(v)deg_{G_{1}+G_{2}}^{2}(v)]+2\sum_{uv\in E}\xi (u)\xi (v)deg_{G_{1}+G_{2}}(u)deg_{G_{1}+G_{2}}(v)=\underset{K_{1}}{\underset{︸}{\sum_{uv\in E_{1}}[\xi^{2}(u)deg_{G_{1}+G_{2}}^{2}(u)+\xi^{2}(v)deg_{G_{1}+G_{2}}^{2}(v)]}}+\underset{K_{2}}{\underset{︸}{\sum_{uv\in E_{2}}[\xi^{2}(u)deg_{G_{1}+G_{2}}^{2}(u)+\xi^{2}(v)deg_{G_{1}+G_{2}}^{2}(v)]}}+\underset{K_{3}}{\underset{︸}{\sum_{u\in V_{1},v\in V_{2}}[\xi^{2}(u)deg_{G_{1}+G_{2}}^{2}(u)+\xi^{2}(v)deg_{G_{1}+G_{2}}^{2}(v)]}}+2FZI_{2}(G_{1}+G_{2}).$$

$$\text{Now }K_{1}=\sum_{uv\in E_{1}}[\xi^{2}(u)deg_{G_{1}+G_{2}}^{2}(u)+\xi^{2}(v)deg_{G_{1}+G_{2}}^{2}(v)]\leq \sum_{uv\in E_{1}}[\xi_{1}^{2}(u){\left\{deg_{G_{1}}(u)+n_{2}\xi_{1}(u)\right\}}^{2}+\xi_{1}^{2}(v){\left\{deg_{G_{1}}(v)+n_{2}\xi_{1}(v)\right\}}^{2}]\leq 2\sum_{uv\in E_{1}}{\left(n_{2}+\Delta_{1}\right)}^{2}=2m_{1}{\left(n_{2}+\Delta_{1}\right)}^{2}.\text{Again, }K_{1}=\sum_{uv\in E_{1}}[\xi^{2}(u)deg_{G_{1}+G_{2}}^{2}(u)+\xi^{2}(v)deg_{G_{1}+G_{2}}^{2}(v)]\geq \sum_{uv\in E_{1}}[\xi_{1}^{2}(u)deg_{G_{1}}^{2}(u)+\xi_{1}^{2}(v)deg_{G_{1}}^{2}(v)]\geq 2\sum_{uv\in E_{1}}[\xi_{1}(u)\xi_{1}(v)deg_{G_{1}}(u)deg_{G_{1}}(v)]=2FZI_{2}(G_{1}).$$

Therefore, $2FZI_{2}(G_{1})\leq K_{1}\leq 2m_{1}{\left(n_{2}+\Delta_{1}\right)}^{2}$.

Similarly, $2FZI_{2}(G_{2})\leq K_{2}\leq 2m_{2}{\left(n_{1}+\Delta_{2}\right)}^{2}$.

$$\text{Also, }K_{3}=\sum_{u\in V_{1},v\in V_{2}}[\xi^{2}(u)deg_{G_{1}+G_{2}}^{2}(u)+\xi^{2}(v)deg_{G_{1}+G_{2}}^{2}(v)]\leq \sum_{u\in V_{1},v\in V_{2}}[\xi_{1}^{2}(u){\left\{deg_{G_{1}}(u)+n_{2}\xi_{1}(u)\right\}}^{2}+\xi_{2}^{2}(v){\left\{deg_{G_{2}}(v)+n_{1}\xi_{2}(v)\right\}}^{2}]\leq n_{2}FZI_{1}(G_{1})+n_{1}FZI_{1}(G_{2})+n_{1}n_{2}(n_{1}^{2}+n_{2}^{2})+2n_{1}n_{2}(n_{2}\Delta_{1}+n_{1}\Delta_{2})\text{and }K_{3}=\sum_{u\in V_{1},v\in V_{2}}[\xi^{2}(u)deg_{G_{1}+G_{2}}^{2}(u)+\xi^{2}(v)deg_{G_{1}+G_{2}}^{2}(v)]\geq \sum_{u\in V_{1},v\in V_{2}}[\xi_{1}^{2}(u)deg_{G_{1}}^{2}(u)+\xi_{2}^{2}(v)deg_{G_{2}}^{2}(v)]=n_{2}FZI_{1}(G_{1})+n_{1}FZI_{1}(G_{2}).$$

Therefore, $n_{2}FZI_{1}(G_{1})+n_{1}FZI_{1}(G_{2})\leq K_{3}\leq n_{2}FZI_{1}(G_{1})+n_{1}FZI_{1}(G_{2})+n_{1}n_{2}(n_{1}^{2}+n_{2}^{2})+2n_{1}n_{2}(n_{2}\Delta_{1}+n_{1}\Delta_{2})$.

Using the value of $K_{1},K_{2},K_{3}$ one can get the result easily. □

Theorem 4.4 $FHZI(G_{1}\cup G_{2})\geq 2[FZI_{2}(G_{1}\cup G_{2})+(m_{1}-m)\delta_{1}+(m_{2}-m)\delta_{2}+m\delta ]$ *, where* $m=|E_{1}\cap E_{2}|,\delta =\wedge \{\delta_{1},\delta_{2}\}$ *.*

Proof As $G_{1}\cup G_{2}$ is union of $G_{1}$ and $G_{2}$, then for u,v∈V

$$\xi (u)=\left\{ \begin{array}{c} \xi_{1}(u),\;u\in V_{1}\setminus V_{2}\\ \xi_{2}(u),\;u\in V_{2}\setminus V_{1}\\ \vee \{\xi_{1}(u),\xi_{2}(u)\},\;u\in V_{1}\cap V_{2} \end{array}\right.$$

$$\text{and }\eta (u,v)=\left\{ \begin{array}{c} \eta_{1}(u,v)\;uv\in E_{1}\setminus E_{2}\\ \eta_{2}(u,v)\;uv\in E_{2}\setminus E_{1}\\ \vee \{\xi_{1}(u,v),\xi_{2}(u,v)\}\;(u,v)\in E_{1}\cap E_{2}\\ 0\;\text{ otherwise.} \end{array}\right.$$

Then,

$$deg_{G_{1}\cup G_{2}}(u)\geq \left\{ \begin{array}{c} deg_{1}(u)\;u\in V_{1}\setminus V_{2}\\ deg_{2}(u)\;u\in V_{2}\setminus V_{1}\\ deg_{1}(u)\;\text{ or }deg_{2}(u)\;u\in V_{1}\cap V_{2} \end{array}\right.$$

$$\text{Now, }FHZI(G_{1}\cup G_{2})=\sum_{uv\in E}{\left[\xi (u)deg_{G_{1}\cup G_{2}}(u)+\xi (v)deg_{G_{1}\cup G_{2}}(v)\right]}^{2}=\sum_{uv\in E}[\xi^{2}(u)deg_{G_{1}\cup G_{2}}^{2}(u)+\xi^{2}(v)deg_{G_{1}\cup G_{2}}^{2}(v)]+2FZI_{2}(G_{1}\cup G_{2})=\sum_{uv\in E_{1}\setminus E_{2}}[\xi^{2}(u)deg_{G_{1}\cup G_{2}}^{2}(u)+\xi^{2}(v)deg_{G_{1}\cup G_{2}}^{2}(v)]+\sum_{uv\in E_{2}\setminus E_{1}}[\xi^{2}(u)deg_{G_{1}\cup G_{2}}^{2}(u)+\xi^{2}(v)deg_{G_{1}\cup G_{2}}^{2}(v)]+\sum_{uv\in E_{1}\cap E_{2}}[\xi^{2}(u)deg_{G_{1}\cup G_{2}}^{2}(u)+\xi^{2}(v)deg_{G_{1}\cup G_{2}}^{2}(v)]+2FZI_{2}(G_{1}\cup G_{2})\geq \sum_{uv\in E_{1}\setminus E_{2}}[\xi_{1}^{2}(u)deg_{G_{1}}^{2}(u)+\xi_{1}^{2}(v)deg_{G_{1}}^{2}(v)]+\sum_{uv\in E_{2}\setminus E_{1}}[\xi_{2}^{2}(u)deg_{G_{2}}^{2}(u)+\xi_{2}^{2}(v)deg_{G_{2}}^{2}(v)]+\sum_{uv\in E_{1}\cap E_{2}}[\xi^{2}(u)deg_{G_{1}\cup G_{2}}^{2}(u)+\xi^{2}(v)deg_{G_{1}\cup G_{2}}^{2}(v)]+2FZI_{2}(G_{1}\cup G_{2})\geq 2FZI_{2}(G_{1}\cup G_{2})+2(m_{1}-m)\delta_{1}+2(m_{2}-m)\delta_{2}+2m\delta .$$

 □

Theorem 4.5 $FHZI(G_{1}\prod G_{2})\leq 2m_{1}m_{2}\max\{\Delta_{1}^{2},\Delta_{2}^{2}\}+2FZI_{2}(G_{1}\prod G_{2})$ *.*

Proof As $G_{1}\prod G_{2}$ is direct product graph of $G_{1}$ and $G_{2}$, then, $\xi (u,v)=\xi_{1}(u)\wedge \xi_{2}(v)$ and $\eta ((u_{1},v_{1})(u_{2},v_{2}))=\eta_{1}(u_{1}u_{2})\wedge \eta_{2}(v_{1}v_{2})$. Hence,

$$deg_{G_{1}\prod G_{2}}(u,v)=\sum_{(u_{1},v_{1})\in V}\eta ((u,v)(u_{1},v_{1}))=\sum_{(u_{1},v_{1})\in V}\eta_{1}(u,u_{1})\wedge \eta_{2}(v,v_{1})\leq deg_{G_{1}}(u)\wedge deg_{G_{2}}(v)\leq \max\{\Delta_{1},\Delta_{2}\}.$$

Therefore,

$$FHZI(G_{1}\prod G_{2})=\sum_{(u_{1},v_{1})(u_{2},v_{2})\in E}{\left[\xi (u_{1},v_{1})deg_{G_{1}\prod G_{2}}(u_{1},v_{1})+\xi (u_{2},v_{2})deg_{G_{1}\prod G_{2}}(u_{2},v_{2})\right]}^{2}=\sum_{(u_{1},v_{1})(u_{2},v_{2})\in E}[\xi^{2}(u_{1},v_{1})deg_{G_{1}\prod G_{2}}^{2}(u_{1},v_{1})+\xi^{2}(u_{2},v_{2})deg_{G_{1}\prod G_{2}}^{2}(u_{2},v_{2})]+2FZI_{2}(G_{1}\prod G_{2})\leq 2m_{1}m_{2}\max\{\Delta_{1}^{2},\Delta_{2}^{2}\}+2FZI_{2}(G_{1}\prod G_{2}).$$

 □

Theorem 4.6 $FHZI(G_{1}•G_{2})\leq 2m_{2}FZI_{1}(G_{1})+2FZI_{2}(G_{1}•G_{2})+8n_{1}m_{2}\Delta_{2}(\Delta_{1}+\Delta_{2})+2m_{1}m_{2}{\left(\Delta_{1}+2\Delta_{2}\right)}^{2}$ *.*

Proof As $G_{1}•G_{2}$ is semi-strong product graph of $G_{1}$ and $G_{2}$, then, $\xi (u,v)=\xi_{1}(u)\wedge \xi_{2}(v)$ and

$$\eta ((u_{1},v_{1})(u_{2},v_{2}))=\left\{ \begin{array}{c} \eta_{1}(u_{1}u_{2})\wedge \eta_{2}(v_{1}v_{2})\;\text{ if }u_{1}u_{2}\in E_{1}\text{ and }v_{1}v_{2}\in E_{2}\\ \xi_{1}(u)\wedge \eta_{2}(v_{1}v_{2})\;\text{ if }u_{1}=u_{2}=u\in V_{1}\text{ and }v_{1}v_{2}\in E_{2}. \end{array}\right.$$

Hence,

$$deg_{G_{1}•G_{2}}(u,v)=\sum_{uu_{1}\in E_{1},vv_{1}\in E_{2}}\eta ((u,v)(u_{1},v_{1}))+\sum_{vv_{1}\in E_{2}}\eta ((u,v)(u,v_{1}))=\sum_{uu_{1}\in E_{1},vv_{1}\in E_{2}}\eta_{1}(uu_{1})\wedge \eta_{2}(vv_{1})+\sum_{vv_{1}\in E_{2}}\xi_{1}(u)\wedge \eta_{2}(vv_{1})\leq \sum_{uu_{1}\in E_{1}}\eta_{1}(uu_{1})+\sum_{vv_{1}\in E_{2}}\eta_{2}(vv_{1})+\sum_{vv_{1}\in E_{2}}\eta_{2}(vv_{1})=deg_{G_{1}}(u)+2deg_{G_{2}}(v).$$

Therefore,

$$FHZI(G_{1}•G_{2})=\sum_{(u_{1},v_{1})(u_{2},v_{2})\in E}{\left[\xi (u_{1},v_{1})deg_{G_{1}•G_{2}}(u_{1},v_{1})+\xi (u_{2},v_{2})deg_{G_{1}•G_{2}}(u_{2},v_{2})\right]}^{2}=\sum_{(u_{1},v_{1})(u_{2},v_{2})\in E}[\xi^{2}(u_{1},v_{1})deg_{G_{1}•G_{2}}^{2}(u_{1},v_{1})+\xi^{2}(u_{2},v_{2})deg_{G_{1}•G_{2}}^{2}(u_{2},v_{2})]+2FZI_{2}(G_{1}•G_{2})=\underset{K_{1}}{\underset{︸}{\sum_{u_{1}u_{2}\in E_{1},v_{1}v_{2}\in E_{2}}[\xi^{2}(u_{1},v_{1})deg_{G_{1}•G_{2}}^{2}(u_{1},v_{1})+\xi^{2}(u_{2},v_{2})deg_{G_{1}•G_{2}}^{2}(u_{2},v_{2})]}}+\underset{K_{2}}{\underset{︸}{\sum_{u\in V_{1},v_{1}v_{2}\in E_{2}}[\xi^{2}(u,v_{1})deg_{G_{1}•G_{2}}^{2}(u,v_{1})+\xi^{2}(u,v_{2})deg_{G_{1}•G_{2}}^{2}(u,v_{2})]}}+2FZI_{2}(G_{1}•G_{2}).$$

Now,

$$K_{1}=\sum_{u_{1}u_{2}\in E_{1},v_{1}v_{2}\in E_{2}}[\xi^{2}(u_{1},v_{1})deg_{G_{1}•G_{2}}^{2}(u_{1},v_{1})+\xi^{2}(u_{2},v_{2})deg_{G_{1}•G_{2}}^{2}(u_{2},v_{2})]\leq \sum_{u_{1}u_{2}\in E_{1},v_{1}v_{2}\in E_{2}}[\xi^{2}(u_{1},v_{1}){\left\{deg_{G_{1}}(u_{1})+2deg_{G_{2}}(v_{1})\right\}}^{2}+\xi^{2}(u_{2},v_{2}){\left\{deg_{G_{1}}(u_{2})+2deg_{G_{2}}(v_{2})\right\}}^{2}]\leq 2\sum_{u_{1}u_{2}\in E_{1},v_{1}v_{2}\in E_{2}}{\left[\Delta_{1}+2\Delta_{2}\right]}^{2}=2m_{1}m_{2}{\left[\Delta_{1}+2\Delta_{2}\right]}^{2}.$$

Again,

$$K_{2}=\sum_{u\in V_{1},v_{1}v_{2}\in E_{2}}[\xi^{2}(u,v_{1})deg_{G_{1}•G_{2}}^{2}(u,v_{1})+\xi^{2}(u,v_{2})deg_{G_{1}•G_{2}}^{2}(u,v_{2})]\leq \sum_{u\in V_{1},v_{1}v_{2}\in E_{2}}[\xi^{2}(u,v_{1}){\left\{deg_{G_{1}}(u)+2deg_{G_{2}}(v_{1})\right\}}^{2}+\xi^{2}(u,v_{2}){\left\{deg_{G_{1}}(u)+2deg_{G_{2}}(v_{2})\right\}}^{2}]\leq \sum_{u\in V_{1},v_{1}v_{2}\in E_{2}}[\{\xi^{2}(u)deg_{G_{1}}^{2}(u)+4deg_{G_{2}}^{2}(v_{1})+4deg_{G_{1}}(u)deg_{G_{2}}(v_{1})\}+\{\xi^{2}(u)deg_{G_{1}}^{2}(u)+4deg_{G_{2}}^{2}(v_{2})+4deg_{G_{1}}(u)deg_{G_{2}}(v_{2})\}]\leq 2\sum_{u\in V_{1},v_{1}v_{2}\in E_{2}}\{\xi^{2}(u)deg_{G_{1}}^{2}(u)\}+\sum_{u\in V_{1},v_{1}v_{2}\in E_{2}}[\{4deg_{G_{2}}^{2}(v_{1})+4deg_{G_{1}}(u)deg_{G_{2}}(v_{1})+4deg_{G_{2}}^{2}(v_{2})+4deg_{G_{1}}(u)deg_{G_{2}}(v_{2})\}]\leq 2m_{2}FZI_{1}(G_{1})+8\sum_{u\in V_{1},v_{1}v_{2}\in E_{2}}\Delta_{2}(\Delta_{1}+\Delta_{2})=2m_{2}FZI_{1}(G_{1})+8n_{1}m_{2}\Delta_{2}(\Delta_{1}+\Delta_{2}).$$

Hence,

$$FHZI(G_{1}•G_{2})=K_{1}+K_{2}+2FZI_{2}(G_{1}•G_{2})\leq 2m_{2}FZI_{1}(G_{1})+2FZI_{2}(G_{1}•G_{2})+8n_{1}m_{2}\Delta_{2}(\Delta_{1}+\Delta_{2})+2m_{1}m_{2}{\left(\Delta_{1}+2\Delta_{2}\right)}^{2}.$$

 □

Theorem 4.7 $FHZI(G_{1}⊗G_{2})\leq 8m_{2}FZI_{1}(G_{1})+8m_{1}FZI_{1}(G_{2})+2FZI_{2}(G⊗G_{2})+4(n_{1}m_{2}+n_{2}m_{1}+2m_{1}m_{2}){\left(\Delta_{1}+\Delta_{2}\right)}^{2}$ *.*

Proof As $G_{1}⊗G_{2}$ is strong product graph of $G_{1}$ and $G_{2}$, then, $\xi (u,v)=\xi_{1}(u)\wedge \xi_{2}(v)$ and

$$\eta ((u_{1},v_{1})(u_{2},v_{2}))=\left\{ \begin{array}{c} \eta_{1}(u_{1}u_{2})\wedge \eta_{2}(v_{1}v_{2})\;\text{ if }u_{1}u_{2}\in E_{1}\text{ and }v_{1}v_{2}\in E_{2}\\ \xi_{1}(u)\wedge \eta_{2}(v_{1}v_{2})\;\text{ if }u_{1}=u_{2}=u\in V_{1}\text{ and }v_{1}v_{2}\in E_{2}\\ \xi_{2}(v)\wedge \eta_{1}(u_{1}u_{2})\;\text{ if }u_{1}u_{2}\in E_{1}\text{ and }v_{1}=v_{2}=v\in V_{2} \end{array}\right.$$

Hence,

$$deg_{G_{1}⊗G_{2}}(u,v)=\sum_{uu_{1}\in E_{1},vv_{1}\in E_{2}}\eta ((u,v)(u_{1},v_{1}))+\sum_{vv_{1}\in E_{2}}\eta ((u,v)(u,v_{1}))+\sum_{uu_{1}\in E_{1}}\eta ((u,v)(u_{1},v))=\sum_{uu_{1}\in E_{1},vv_{1}\in E_{2}}\eta_{1}(uu_{1})\wedge \eta_{2}(vv_{1})+\sum_{vv_{1}\in E_{2}}\xi_{1}(u)\wedge \eta_{2}(vv_{1})+\sum_{uu_{1}\in E_{1}}\xi_{2}(v)\wedge \eta_{1}(uu_{1})\leq \sum_{uu_{1}\in E_{1}}\eta_{1}(uu_{1})+\sum_{vv_{1}\in E_{2}}\eta_{2}(vv_{1})+\sum_{vv_{1}\in E_{2}}\eta_{2}(vv_{1})+\sum_{uu_{1}\in E_{1}}\eta_{1}(uu_{1})=2[deg_{G_{1}}(u)+deg_{G_{2}}(v)].$$

Therefore,

$$FHZI(G_{1}⊗G_{2})=\sum_{(u_{1},v_{1})(u_{2},v_{2})\in E}{\left[\xi (u_{1},v_{1})deg_{G_{1}⊗G_{2}}(u_{1},v_{1})+\xi (u_{2},v_{2})deg_{G_{1}⊗G_{2}}(u_{2},v_{2})\right]}^{2}=\sum_{(u_{1},v_{1})(u_{2},v_{2})\in E}[\xi^{2}(u_{1},v_{1})deg_{G_{1}⊗G_{2}}^{2}(u_{1},v_{1})+\xi^{2}(u_{2},v_{2})deg_{G_{1}⊗G_{2}}^{2}(u_{2},v_{2})]+2FZI_{2}(G_{1}⊗G_{2})=\underset{K_{1}}{\underset{︸}{\sum_{u_{1}u_{2}\in E_{1},v_{1}v_{2}\in E_{2}}[\xi^{2}(u_{1},v_{1})deg_{G_{1}⊗G_{2}}^{2}(u_{1},v_{1})+\xi^{2}(u_{2},v_{2})deg_{G_{1}⊗G_{2}}^{2}(u_{2},v_{2})]}}+\underset{K_{2}}{\underset{︸}{\sum_{u\in V_{1},v_{1}v_{2}\in E_{2}}[\xi^{2}(u,v_{1})deg_{G_{1}⊗G_{2}}^{2}(u,v_{1})+\xi^{2}(u,v_{2})deg_{G_{1}⊗G_{2}}^{2}(u,v_{2})]}}+\underset{K_{3}}{\underset{︸}{\sum_{u_{1}u_{2}\in E_{1},v\in V_{2}}[\xi^{2}(u_{1},v)deg_{G_{1}⊗G_{2}}^{2}(u_{1},v)+\xi^{2}(u_{2},v)deg_{G_{1}⊗G_{2}}^{2}(u_{2},v)]}}+2FZI_{2}(G_{1}⊗G_{2})$$

Now,

$$K_{1}=\sum_{u_{1}u_{2}\in E_{1},v_{1}v_{2}\in E_{2}}[\xi^{2}(u_{1},v_{1})deg_{G_{1}⊗G_{2}}^{2}(u_{1},v_{1})+\xi^{2}(u_{2},v_{2})deg_{G_{1}⊗G_{2}}^{2}(u_{2},v_{2})]\leq 4\sum_{u_{1}u_{2}\in E_{1},v_{1}v_{2}\in E_{2}}[\xi^{2}(u_{1},v_{1}){\left\{deg_{G_{1}}(u_{1})+deg_{G_{2}}(v_{1})\right\}}^{2}+\xi^{2}(u_{2},v_{2}){\left\{deg_{G_{1}}(u_{2})+deg_{G_{2}}(v_{2})\right\}}^{2}]\leq 8m_{1}m_{2}{\left(\Delta_{1}+\Delta_{2}\right)}^{2}.$$

Again,

$$K_{2}=\sum_{u\in V_{1},v_{1}v_{2}\in E_{2}}[\xi^{2}(u,v_{1})deg_{G_{1}⊗G_{2}}^{2}(u,v_{1})+\xi^{2}(u,v_{2})deg_{G_{1}⊗G_{2}}^{2}(u,v_{2})]\leq 4\sum_{u\in V_{1},v_{1}v_{2}\in E_{2}}[\xi^{2}(u,v_{1}){\left\{deg_{G_{1}}(u)+deg_{G_{2}}(v_{1})\right\}}^{2}+\xi^{2}(u,v_{2}){\left\{deg_{G_{1}}(u)+deg_{G_{2}}(v_{2})\right\}}^{2}]\leq 4\sum_{u\in V_{1},v_{1}v_{2}\in E_{2}}[\xi^{2}(u,v_{1})\{deg_{G_{1}}^{2}(u)+deg_{G_{2}}^{2}(v_{1})+deg_{G_{1}}(u)deg_{G_{2}}(v_{1})\}+\xi^{2}(u,v_{2})\{deg_{G_{1}}^{2}(u)+deg_{G_{2}}^{2}(v_{2})+deg_{G_{1}}(u)deg_{G_{2}}(v_{2})\}]=4\sum_{u\in V_{1},v_{1}v_{2}\in E_{2}}[deg_{G_{2}}^{2}(v_{1})+2deg_{G_{1}}(u)deg_{G_{2}}(v_{1})+deg_{G_{2}}^{2}(v_{2})]+8\sum_{u\in V_{1},v_{1}v_{2}\in E_{2}}\xi^{2}(u,v_{1})deg_{G_{1}}^{2}(u)\leq 8m_{2}FZI_{1}(G_{1})+4n_{1}m_{2}(\Delta_{1}^{2}+\Delta_{2}^{2}+2\Delta_{1}\Delta_{2})=8m_{2}FZI_{1}(G_{1})+4n_{1}m_{2}{\left(\Delta_{1}+\Delta_{2}\right)}^{2}.$$

Similarly,

$$K_{3}=\sum_{u_{1}u_{2}\in E_{1},v\in V_{2}}[\xi^{2}(u_{1},v)deg_{G_{1}⊗G_{2}}^{2}(u_{1},v)+\xi^{2}(u_{2},v)deg_{G_{1}⊗G_{2}}^{2}(u_{2},v)]\leq 8m_{1}FZI_{1}(G_{2})+4n_{2}m_{1}{\left(\Delta_{1}+\Delta_{2}\right)}^{2}.$$

Hence,

$$FHZI(G⊗G_{2})=K_{1}+K_{2}+K_{3}+2FZI_{2}(G⊗G_{2})\leq 8m_{1}m_{2}{\left(\Delta_{1}+\Delta_{2}\right)}^{2}+8m_{2}FZI_{1}(G_{1})+4n_{1}m_{2}{\left(\Delta_{1}+\Delta_{2}\right)}^{2}+8m_{1}FZI_{1}(G_{2})+4n_{2}m_{1}{\left(\Delta_{1}+\Delta_{2}\right)}^{2}+2FZI_{2}(G⊗G_{2})=8m_{2}FZI_{1}(G_{1})+8m_{1}FZI_{1}(G_{2})+2FZI_{2}(G⊗G_{2})+4(n_{1}m_{2}+n_{2}m_{1}+2m_{1}m_{2}){\left(\Delta_{1}+\Delta_{2}\right)}^{2}.$$

 □

## 5 Application

Crime is a pervasive global issue that reflects a grim aspect of society. Different countries delineate crime in various ways, but acts of violence consistently pose a significant threat to societal well-being. In India, the recording of crimes dates back to the British Raj, with the National Crime Records Bureau, operating under the Ministry of Home Affairs, compiling annual statistics. As of 2020, there were 66 lakh admissible offences, comprising 42.5 lakh Indian Penal Code (IPC) offences and 23.5 lakh Special and Local Law (SLL) offences. This represents a notable annual increase in crime of 28.02%. A substantial portion of these registered crimes—more than one-fifth—were categorized as crimes affecting the human body, encompassing violent acts such as murder, kidnapping, assault and death by negligence. In this section, we focus on the analysis of the crime of “murder” across various states in India.

In this section, we examine the crime of “murder” across different states in India. Specifically, we explore the extent to which the incidence of this crime in a state is influenced by criminals from its neighboring states as well as within its own jurisdiction.

### 5.1 Graph construction

Our objective is to examine the impact of crime within a state, both from neighboring states and within its own jurisdiction. In this analysis, each state is represented as a vertex and an edge is established between neighboring states. States that are not immediate neighbors exert considerably less influence on crime and therefore, no edges are considered for such pairs.

### 5.2 Assigning membership values

The vertex membership value of a state (*S*) is contingent upon the number of murders occurring per ten lakh population (n(S)). A high value of n(S) signifies a high crime rate in state *S*, while a lower value indicates a lower crime rate. Therefore, the vertex membership value of a state $S_{i}$ is defined as:

$$\xi (S_{i})=\frac{n(S_{i})}{\vee \{n(S):S\text{ is a state of India}\}}.$$

It's important to note that a higher membership value of a state indicates lower safety for its people, while a lower membership value signifies higher safety. In many cases, perpetrators seek refuge in neighboring states, making the study of this type of crime imperative. Consequently, edges are established between neighboring states. The edge membership value is contingent upon the influence relationship between states regarding the occurrence of crime. Therefore, the edge membership value for an edge $S_{i}S_{j}$ is defined as:

$$\eta (S_{i}S_{j})=\min\{\xi (S_{i}),\xi (S_{j})\}.$$

**Figure 2:**
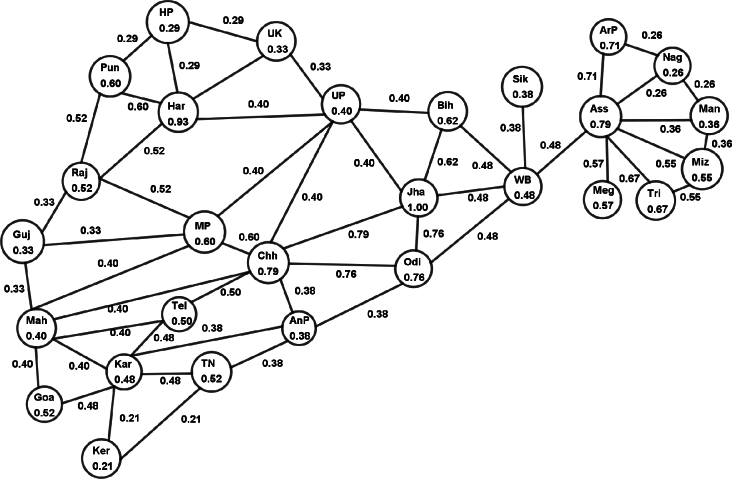
Model fuzzy graph *G*.

Note that $\eta (S_{i}S_{j})$ represents the maximum effect of the crime for $S_{i}$ by the state's criminal $S_{j}$ or $S_{j}$ by the criminal of the state $S_{i}$. The higher membership value of an edge represents the lower safety for the people of the two states. The lower membership value of an edge represents the higher safety for the people of the two states. The set of vertices and vertex membership values are listed in Table 1 and the set of edges and edge membership values are listed in Table 2. Hence the model FG *G* is shown in Fig. 2.

**Table 1:** Vertex membership value.

State name	Vertex name	Number of murder per ten Lakh population	Vertex membership value
Andhra Pradesh	AnP	1.6	0.38
Arunachal Pradesh	ArP	3.0	0.71
Assam	Ass	3.3	0.79
Bihar	Bih	2.6	0.62
Chhattisgarh	Chh	3.3	0.79
Goa	Goa	2.2	0.52
Gujarat	Guj	1.4	0.33
Haryana	Har	3.9	0.93
Himachal Pradesh	HP	1.2	0.29
Jharkhand	Jha	4.2	1.00
Karnataka	Kar	2.0	0.48
Kerala	Ker	0.9	0.21
Madhya Pradesh	MP	2.5	0.60
Maharashtra	Mah	1.7	0.40
Manipur	Man	1.5	0.36
Meghalaya	Meg	2.4	0.57
Mizoram	Miz	2.3	0.55
Nagaland	Nag	1.1	0.26
Odisha	Odi	3.2	0.76
Punjab	Pun	2.5	0.60
Rajasthan	Raj	2.2	0.52
Sikkim	Sik	1.6	0.38
Tamil Nadu	TN	2.2	0.52
Telangana	Tel	2.1	0.50
Tripura	Tri	2.8	0.67
Uttar Pradesh	UP	1.7	0.40
Uttarakhand	UK	1.4	0.33
West Bengal	WB	2.0	0.48

**Table 2:** Edge membership value.

Edge name	Edge membership value	Edge name	Edge membership value	Edge name	Edge membership value	Edge name	Edge membership value
AnP-Chh	0.38	AnP-Kar	0.38	AnP-Odi	0.38	AnP-TN	0.38
AnP-Tel	0.38	ArP-Ass	0.71	ArP-Nag	0.26	Ass-Man	0.36
Ass-Meg	0.57	Ass-Miz	0.55	Ass-Nag	0.26	Ass-Tri	0.67
Ass-WB	0.48	Bih-Jha	0.62	Bih-UP	0.40	Bih-WB	0.48
Chh-Jha	0.79	Chh-MP	0.6	Chh-Mah	0.4	Chh-Odi	0.76
Chh-Tel	0.50	Chh-UP	0.40	Goa-Kar	0.48	Goa-Mah	0.40
Guj-MP	0.33	Guj-Mah	0.33	Guj-Raj	0.33	Har-HP	0.29
Har-Pun	0.60	Har-Raj	0.52	Har-UP	0.40	Har-UK	0.33
HP-Pun	0.29	HP-UK	0.29	Jha-Odi	0.76	Jha-UP	0.40
Jha-WB	0.48	Kar-Ker	0.21	Kar-Mah	0.40	Kar-TN	0.48
Kar-Tel	0.48	Ker-TN	0.21	MP-Mah	0.40	MP-Raj	0.52
MP-UP	0.40	Mah-Tel	0.40	Man-Miz	0.36	Man-Nag	0.26
Miz-Tri	0.55	Odi-WB	0.48	Pun-Raj	0.52	Raj-UP	0.40
Sik-WB	0.38	UP-UK	0.33

### 5.3 Method to determine the crime affected for a state by the criminal of its neighbor states and itself

In our investigation of the impact of crime on a state by criminals from its neighboring states and within its own jurisdiction, we adhere to the following steps:

**Step 1:** For each state *S*, we construct an induced fuzzy subgraph of *G*, comprising the vertices *S* and its neighboring states. Let G(S) denote the fuzzy subgraph corresponding to the state *S*. It's important to note that the vertex membership and edge membership values in G(S) remain consistent with those in the model FG.

**Step 2:** For each State *S*, the degree of each vertex $S_{i}$ is evaluated by the formula:

$$deg_{G(S)}(S_{i})=\sum_{S_{i}S_{j}\in E(G(S))}\eta (S_{i}S_{j}).$$

In this context, the degree of vertex $S_{i}$ signifies the cumulative impact of crime on $S_{i}$ by criminals from its neighboring states. It's worth noting that this calculation excludes the impact of crime by $S_{i}$ on itself.

**Step 3:** Now, the score of a state is hyper-ZI for the fuzzy subgraph G(S) and which is calculated by the formula:

$$Score(S)=FHZI(G(S))=\sum_{S_{i}S_{j}\in E(G(S))}{\left[\xi_{G(S)}(S_{i})deg_{G(S)}(S_{i})+\xi_{G(S)}(S_{j})deg_{G(S)}(S_{j})\right]}^{2}.$$

The score of a state is closely linked to its neighboring states; when the crime rate of a neighboring state is high, the score of the state in question also increases and vice versa. Additionally, the impact of crime on the state *S* itself is taken into account. Therefore, the score reflects the total extent of crime affecting the state, originating from both its neighboring states and its own jurisdiction.

### 5.4 Illustration

In this section, we have elucidated the methodology for assessing the impact of crime on a state, taking into account both the criminal activities of neighboring states and those within the state itself. To illustrate, we focus on the state of “Madhya Pradesh” as a case study.

**Figure 3:**
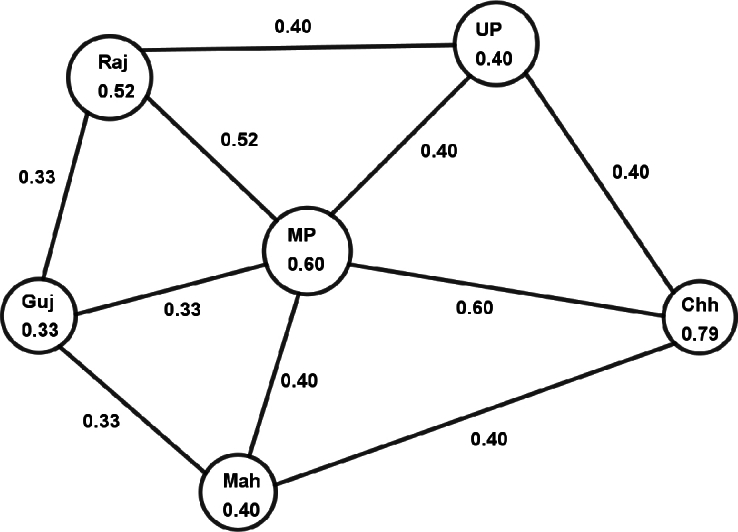
The fuzzy subgraph *G*(*MP*).

**Step 1:** The neighboring vertices of Madhya Pradesh (MP) include Chhattisgarh (Chh), Gujarat (Guj), Maharashtra (Mah), Rajasthan (Raj) and Uttar Pradesh (UP). Thus, the vertex set comprises {Chh, Guj, MP, Mah, Raj, UP}. Consequently, the fuzzy subgraph G(MP) is depicted in Fig. 3.

**Step 2:** Here, the degree of each vertex for the FG G(MP) is calculated by the formula:

$$deg_{G(MP)}(S_{i})=\sum_{S_{i}S_{j}\in E(G(MP))}\eta (S_{i}S_{j}).$$

Using above formula, degree of each vertices is: $deg_{G(MP)}(Chh)=1.40$, $deg_{G(MP)}(Guj)=1.00$, $deg_{G(MP)}(MP)=2.26$, $deg_{G(MP)}(Mah)=1.14$, $deg_{G(MP)}(Raj)=1.26$, $deg_{G(MP)}(UP)=1.21$.

**Step 3:** Now, the score of the state Madhya Pradesh is calculated by the formula:

$$Score(MP)=\sum_{S_{i}S_{j}\in E(G(MP))}{\left[\xi_{G(MP)}(S_{i})deg_{G(MP)}(S_{i})+\xi_{G(MP)}(S_{j})deg_{G(MP)}(S_{j})\right]}^{2}.$$

The score of Madhya Pradesh is determined to be 27.45267. Likewise, the score for each state can be calculated accordingly. Table 3 presents the score values for all states.

**Table 3:** Score value of each vertex for different topological indices.

Vertex name	Hyper-ZI for FGs (Hyper-ZIF)	Hyper-ZI for crisp graphs (Hyper-ZIC)	First ZI for FGs (First-ZIF)	F-index for FGs (FF-index)
AnP	23.27	412	2.01	1.93
ArP	3.66	48	0.55	0.40
Ass	82.23	712	5.04	12.01
Bih	16.54	136	1.71	2.15
Chh	63.53	678	4.17	7.80
Goa	1.96	48	0.25	0.10
Guj	4.73	136	0.48	0.29
Har	38.02	500	2.81	4.52
HP	7.19	136	0.82	0.80
Jha	107.04	500	7.76	18.34
Kar	20.30	610	1.35	1.14
Ker	0.86	48	0.12	0.04
MP	27.45	500	2.02	2.16
Mah	27.68	610	2.04	1.86
Man	5.65	136	0.61	0.47
Meg	0.60	4	0.15	0.06
Miz	14.20	136	1.44	1.47
Nag	7.22	136	0.84	0.72
Odi	57.31	256	4.99	8.93
Pun	13.09	136	1.36	1.49
Raj	29.65	412	2.38	2.73
Sik	0.11	4	0.03	0.00
TN	3.30	136	0.33	0.17
Tel	16.00	340	1.29	1.07
Tri	7.51	48	0.96	0.81
UP	62.75	856	4.86	6.57
UK	5.02	136	0.56	0.46
WB	26.59	170	3.07	4.45

It's noteworthy that the state of Sikkim has the lowest score value, indicating that it experiences the least impact from the criminal activities of its neighboring states as well as within its own jurisdiction. Conversely, for the state of Jharkhand, the highest score value implies that it faces the greatest impact from the criminal activities of its neighboring states and within its own jurisdiction.

### 5.5 Comparative analysis

To compare, we consider other topological indices: the first ZI for FGs, the F-index for FGs and the hyper-ZI for crisp graphs, to assess the impact of crime on a state by the criminals of its neighboring states and within its own jurisdiction. It's important to note that topological indices defined solely for crisp graphs are independent of the nature of the vertex or its neighboring vertices, meaning they do not consider the number of crimes in any state or its neighbors. Consequently, these indices cannot accurately predict actual crime rates. Therefore, incorporating fuzziness is essential for making such considerations.

The hyper-ZI for FGs takes into account not only the number of crimes in each state but also the total number of crimes in neighboring states for each state. As a result, this index consistently yields realistic results compared to other existing indices. The scores of each state for these indices are presented in Table 3. As shown in the table, each index identifies “Sikkim” as having the lowest score, indicating the lowest impact of crime by the criminals of its neighboring state and itself. All indices, except the hyper-ZI for crisp graphs, identify “Jharkhand” as having the highest score, indicating the highest impact of crime by the criminals of its neighboring state and itself. However, the hyper-ZI defined for crisp graphs assigns the same score value to different states. If decision-makers were to rely solely on indices for crisp graphs, they would be unable to distinguish between states. Therefore, it is imperative to introduce topological indices defined for FGs.

**Figure 4:**
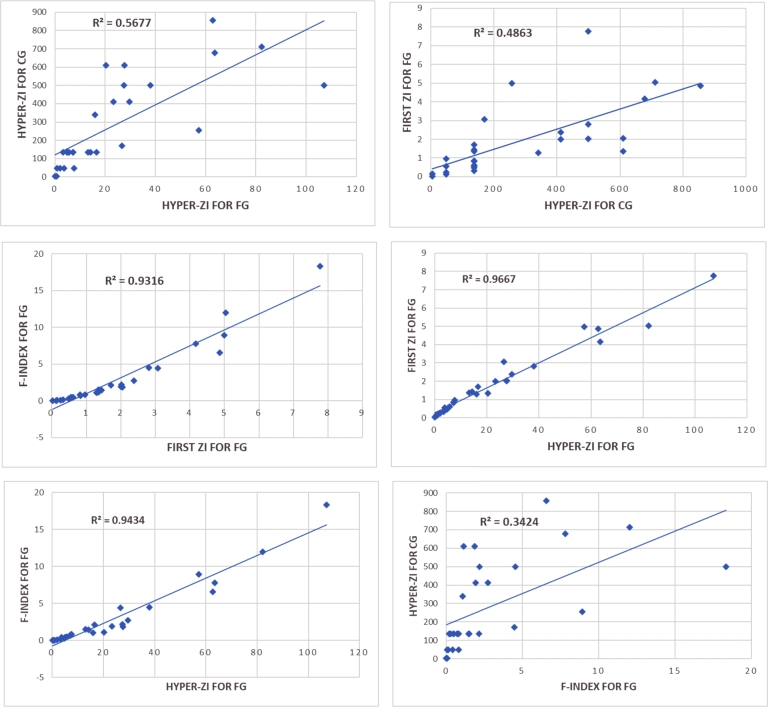
Linear curves fitting among the score values obtained by different indices.

We also fit linear curves among the score values obtained by these indices (See Fig. 4). The relationship between these indices with respect to the scores is shown below:

 Hyper-ZIC =6.8402(Hyper-ZIF)+120.48,R=0.7535, First-ZIF =0.0053(Hyper-ZIC)+0.406,R=0.6972, FF-index =2.1783(First-ZIF)−1.2407,R=0.9652, Hyper-ZIF =14.127(First-ZIF)−3.201,R=0.9832, FF-index =0.1526(Hyper-ZIF)−0.7074,R=0.9713 and  Hyper-ZIC =33.815(FF-index)+184.85,R=0.5851.

The correlation coefficient value (*R*) indicates that the score derived from the hyper-ZI for FGs is strongly correlated with the scores obtained from the first ZI and F-index for FGs. However, it exhibits a weaker correlation with the score obtained from the hyper-ZI for crisp graphs.

## 6 Conclusion

The hyper-ZI plays a crucial role in various fields including chemical graph theory, spectral graph theory, network theory, molecular chemistry and FG (FG) theory. In this study, we have defined the hyper-ZI specifically for FGs and explored its properties across various types of FGs, such as paths, cycles, stars, complete FGs and partial fuzzy subgraphs. Notably, we have demonstrated that the value of this index remains consistent for isomorphic FGs. Furthermore, we have established intriguing relationships between the second ZI and the hyper-ZI for FGs and determined the bounds of this index for operations including Cartesian product, composition, join, union, strong product, semi-strong product and direct product of two FGs. Additionally, we have applied the hyper-ZI to analyze the crime of “Murder” across states in India and compared its performance with three other topological indices: the hyper-ZI for crisp graphs, the first ZI for FGs and the F-index for FGs. Our findings suggest that while the hyper-ZI for FGs, the first ZI for FGs and the F-index for FGs yield similar results, the hyper-ZI for FGs provides more realistic results compared to its crisp counterpart in detecting crime in India. Specifically, we have identified that the state of Sikkim experiences the lowest impact from crime, while Jharkhand faces the highest impact from criminal activities of both neighboring states and within its own jurisdiction.

Moving forward, the future scopes of this research include:

(i) While this article focuses on establishing the maximal *n*-vertex FG concerning the hyper-ZI, determining the *n*-vertex FG with the minimum hyper-ZI remains an open question.

(ii) The investigation into which *n*-vertex tree (fuzzy) exhibits the minimum or maximum hyper-ZI is an area for further research.

(iii) Similarly, identifying which *n*-vertex unicyclic graph (fuzzy) has the minimum or maximum hyper-ZI presents an opportunity for future exploration.

## Ethical approval

This article does not contain any studies with human participants or animals performed by any of the authors.

## CRediT authorship contribution statement

**Sk Rabiul Islam:** Writing – review & editing, Writing – original draft, Visualization, Validation, Methodology, Investigation, Formal analysis, Data curation, Conceptualization. **Bandar Bin Mohsin:** Project administration. **Madhumangal Pal:** Writing – review & editing, Supervision.

## Declaration of Competing Interest

The authors declare that they have no known competing financial interests or personal relationships that could have appeared to influence the work reported in this paper.

## Data Availability

All the data are collected from the website of NATIONAL CRIME RECORDS BUREAU (Govt. of India) https://ncrb.gov.in/en/crime-india.
